# Lysophosphatidic Acid Mediates Activating Transcription Factor 3 Expression Which Is a Target for Post-Transcriptional Silencing by miR-30c-2-3p

**DOI:** 10.1371/journal.pone.0139489

**Published:** 2015-09-29

**Authors:** Ha T. Nguyen, Wei Jia, Aaron M. Beedle, Eileen J. Kennedy, Mandi M. Murph

**Affiliations:** Department of Pharmaceutical and Biomedical Sciences, The University of Georgia, College of Pharmacy, 240 W. Green Street, Athens, Georgia 30602, United States of America; Georgia Regents University, UNITED STATES

## Abstract

Although microRNAs (miRNAs) are small, non-protein-coding entities, they have important roles in post-transcriptional regulation of most of the human genome. These small entities generate fine-tuning adjustments in the expression of mRNA, which can mildly or massively affect the abundance of proteins. Previously, we found that the expression of miR-30c-2-3p is induced by lysophosphatidic acid and has an important role in the regulation of cell proliferation in ovarian cancer cells. The goal here is to confirm that ATF3 mRNA is a target of miR-30c-2-3p silencing, thereby further establishing the functional role of miR-30c-2-3p. Using a combination of bioinformatics, qRT-PCR, immunoblotting and luciferase assays, we uncovered a regulatory pathway between miR-30c-2-3p and the expression of the transcription factor, ATF3. Lysophosphatidic acids triggers the expression of both miR-30c-2-3p and ATF3, which peak at 1 h and are absent 8 h post stimulation in SKOV-3 and OVCAR-3 serous ovarian cancer cells. The 3´-untranslated region (3´-UTR) of ATF3 was a predicted, putative target for miR-30c-2-3p, which we confirmed as a bona-fide interaction using a luciferase reporter assay. Specific mutations introduced into the predicted site of interaction between miR-30c-2-3p and the 3´-UTR of ATF3 alleviated the suppression of the luciferase signal. Furthermore, the presence of anti-miR-30c-2-3p enhanced ATF3 mRNA and protein after lysophosphatidic acid stimulation. Thus, the data suggest that after the expression of ATF3 and miR-30c-2-3p are elicited by lysophosphatidic acid, subsequently miR-30c-2-3p negatively regulates the expression of ATF3 through post-transcriptional silencing, which prevents further ATF3-related outcomes as a consequence of lysophosphatidic acid signaling.

## Introduction

MicroRNAs (miRNAs) are small non-protein-coding RNA molecules approximately 20–24 nucleotides in length that post-transcriptionally regulate gene expression. Originally discovered in 1993 in *C*. *elegans*, miRNAs modulate gene expression by creating fine-tuning adjustments in mRNA expression through their actions as mRNA co-repressors or co-activators [1, 2]. Via non-canonical base pairing with the 3´UTR region of specific mRNAs, a single miRNA molecule is capable of inhibiting the expression of hundreds of different target genes. Although massive gene repression by miRNAs only occurs occasionally, *numerous* mRNA targets are usually modestly repressed by miRNAs [3], further substantiating their role as the genome’s fine-tuners of protein expression. In fact, predictions suggest most of the human genome is regulated by miRNAs.

Several observations made miRNAs incredibly relevant to oncology and cancer research, establishing the moniker, ‘oncomirs’. Foremost, studies show a global decrease in miRNA expression among tumor tissue and cancer cell lines when compared to normal tissue, implying miRNAs function as tumor suppressors [2, 4]. Additionally, profiles of human tumor specimens demonstrate that poorly differentiated tumors are far better classified by miRNA profiles, rather than mRNA [4]. Lastly, among human specimens of invasive epithelial ovarian cancer, the levels of RNA-interference proteins Dicer and Drosha are decreased in 60% and 51%, respectively, and this correlates with poor clinical outcome [5].

We previously identified a miRNA located on chromosome 6q13, miR-30c-2-3p, as a target gene induced by the treatment of ovarian cancer cells with lysophosphatidic acid, which is a lipid mediator abundant in ascites fluid that promotes tumor aggressiveness [6–10]. MiR-30c-2-3p induction resultant from lysophosphatidic acid signaling facilitates the repression of oncogenic mRNA, such as the BCL9 transcript, within 1 hour after stimulation. This suggests the existence of a miRNA-controlled, negative feedback loop that normally serves to regulate oncogenic signaling. This is noteworthy because lysophosphatidic acid-induced profiles correlate with poor prognosis among epithelial ovarian carcinoma [11].

ATF3 is a molecular hub that belongs to the ATF/CREB (cyclic AMP response element-binding) family of transcription factors; furthermore, it plays a role in the promotion of proliferation in malignant cells [12]. It is expressed at nearly undetectable levels in the cell until it is stimulated via serum, cytokines, genotoxic agents or physiological stresses, whereby its transcriptional mRNA is increased, which parallels its protein levels [13]. Interestingly, a gene expression analysis of primary ovarian tumors from patients with either high or low/no depressive symptoms reveals that ATF3 mRNA is significantly amplified among specimens from patients with high levels of depression (**S1 Fig**) [14, 15]. This reinforces the role of ATF3 and intimates an important contextual function among patients with ovarian cancer, especially since stress promotes the progression of ovarian cancer [16].

ATF3 is somewhat enigmatic since it functions as a dualistic transcription factor with both anti- and pro-tumorigenic effects, depending on the cancer subtype. For example, ATF3 suppresses metastasis in bladder cancer but possesses oncogenic effects in cutaneous squamous cell carcinoma [17]. In contrast, among some ovarian cancer cells, ATF3 functions as an apoptosis inducer [18], yet in other ovarian cancer cells it belongs to a profile of worsened outcomes [11].

Other studies have shown that miR-30c-2-3p induces the interference and silencing of transcripts such as HIF2A, X-box binding protein 1, Cyclin E1, BCL9 and an adaptor protein of the NF-κB signaling pathway [6, 19–21]. However, the purpose of this study was to confirm the interaction between ATF3 and miR-30c-2-3p, thereby further establishing the role of miR-30c-2-3p after induction by lysophosphatidic acid in ovarian cancer cells. The data suggests that miR-30c-2-3p inhibits ATF3 mRNA after both are expressed after 1 h treatment of lysophosphatidic acid. Although part of the transcription repression for ATF3 occurs through the recruitment of histone deacetylases to the promoter [22] and miR-494 repression [23], these elements do not account for its complete regulation and are likely context and time specific. This finding is not an unprecedented phenomenon as the literature reports many miRNAs that silence transcription factors [24], but prior to our discovery, nothing was reported about the putative interaction between ATF3 and miR-30c-2.

## Methods

### Cell culture and reagents

Ovarian cancer cell lines, SKOV-3 and OVCAR-3 were obtained from American Type Culture Collection (ATCC, Manassas, VA). All cells were maintained at 37°C, 5% CO_2._ SKOV-3 cells were cultured in Dulbecco’s Modification of Eagles’s medium (DMEM) (Mediatech, Inc., Manassas, VA); OVCAR-3 cells were cultured in RPMI 1640 media (Mediatech). All media were supplemented with 10% fetal bovine serum (Atlanta biological, Flowery Branch, GA). Immortalized ovarian surface epithelial cells, IOSE, were maintained in 1:1 mixture of medium 199 and MCDB 105 (Sigma-Aldrich, St. Louis, MO), supplement with 10% fetal bovine serum (Atlanta Biologicals, Norcross, GA). Lysophosphatidic acid (18:1, 1 oleoyl-2-hydroxy-sn-glycero-3-phosphate) was purchased from Avanti Polar Lipids Inc. (Alabaster, Alabama). For all treatments with lysophosphatidic acid, cells were serum-starved overnight and then treated with serum free media containing lysophosphatidic acid reconstituted in PBS with 1% charcoal-stripped, fatty-acid free, bovine serum albumin (Sigma-Aldrich).

### SiRNA knockdown of ATF3

SKOV-3, OVCAR-3 or IOSE cells were transfected with Dharmacon SMART pools of siRNA (DharmaconRNAi, Lafayett, CO) targeting the ATF3 mRNA using DharmaFECT reagent (DharmaconRNAi) and following the protocol provided by the manufacturer. Control samples were treated with non-targeted siRNA (DharmaconRNAi). A final concentration of 100 nM siRNA was used, and the expression level of ATF3 mRNA were measured 48 h after transfection.

### mRNA isolation and quantitative real-time PCR

Total mRNAs from cells were isolated using Trizol reagent (Thermo Fisher Scientific/Life Technologies, Carlsbad, CA). Total cDNAs and specific cDNAs from miRNAs were generated using iScript^TM^ cDNA Synthesis kit (Bio-Rad, Hercules, CA). Quantitative RT-PCR Taqman primer set for miR-30c-2-3p was purchased from Life Technologies. Real-time PCR was assessed by 7900HT Fast Real-Time PCR system (Applied Biosystems, Foster City, CA) using Power SYBR Green Real-time PCR Master Mix or Taqman Gene Expression Master Mix (also Applied Biosystems). The primers used were based on algorithm-generated sequences from either Primer Bank (http://pga.mgh.harvard.edu/primerbank) or NCBI Primer-BLAST. The primer used were as followed: ATF3 (5´-TCTGCGCTGGAATCAGTCAC-3´ and 5´-GTGGGCCGATGAAGGTTGA-3´), and 18S (5’- AGAAACGGCTACCACATCCA-3´ and 5´- CCCTCCAATGGATCCTCGTT-3´). The reactions were normalized using 18S as housekeeping gene.

### Exogenous expression of synthetic ATF3, miR-30c-2-3p or anti-miR-30c-2-3p

OVCAR-3 or SKOV-3 were plated at approximately 1.5 x 10^5^ cells into 6-well plates and then incubated overnight. Cells were then transfected with miR-30c-2-3p (CUGGGAGAAGGCUGUUUACUCU), anti-miR-30c-2-3p construct or Negative control (Pre-miR miRNA Precursor Negative Control #1)(Life Technologies) where indicated. The negative control does not target any known mRNA within the human transcriptome. The transfection used DharmaFECT 1 (DharmaconRNAi) according to the manufacturer’s instruction. After 48 h transfection, samples were collected for next step of analysis. For ectopic expression of ATF3, cells were transfected with ATF3 overexpression vector, Precision LentiORF (DharmaconRNAi), using Xfect transfection system (Clontech, Mountain View, CA, USA) following manufacturer’s instruction. Approximately 48 h after ATF3 overexpression, cells used in experiments as described.

### Luciferase assay

SKOV-3 cells were seeded in a 12-well plate and incubated overnight. Cells were later transfected with miTarget^TM^ 3´-UTR miRNA Target Clones custom vectors (GeneCopoeia, Rockville, MD) using Xfect™ Transfection reagent (Clontech). The human ATF3 3' UTR sequence was inserted downstream of the secreted Gaussia luciferase (GLuc) reporter gene in a mammalian expression vector with the SV40 promoter and neomycin resistance gene. In the mutated vector, the sequence predicted to interact with miR-30c-2-3p, CTCTTCCGA, in the ATF3 3´-UTR was mutated to GGGGAAAGG and inserted into the vector (GeneCopoeia). After transfection, stable cells were selected with G418 for several weeks prior to the assessment of luciferase. To measure luciferase secretion, approximately 1x10^5^ of stably expressing cells were seeded in a 6-well plate and incubated overnight prior to transfection with miR-30c-2-3p, anti-miR-30c-2-3p or negative miR. After 48 h, media were collected and secreted luciferase was measured in a 96-well plate using Secrete-Pair^TM^ Dual Luminescence Assay kit (GeneCopoeia) with a Synergy 2 Multi-Mode plate reader (BioTek, Winooski, VT). The data presented is the result of experiments performed at least three times. The average luciferase signal is displayed in a bar graph, normalized to the negative-miR control.

### Cell viability and number

SKOV-3 cells were seeded (5 x 10^3^) into a 96-well plate and allowed to attach overnight prior to transfection with anti-miR-30c-2-3p or the ATF3 expression vector where indicated. After 48 h of transfection, cells were fixed with formaldehyde 4% and stained with a Whole Cell Stain (Thermo Fisher Scientific, Waltham, MA) according to the manufacturer’s protocol. Automated fluorescence microscopic images were captured and analyzed using the Cellomics ArraysScan VTI HCS Reader (Thermo Fisher Scientific/Cellomics). Graphs and the statistical calculations were generated using GraphPad Prism (GraphPad Software Inc., La Jolla, CA). Experiments are done with a minimum of quintuplicate samples and at least 5 images per well are taken and incorporated in the data analysis. The experiment was repeated three times. Approximately 10 fields representing 150–750 cells are averaged in the graphs presented. In another experiment, SKOV-3 cells were seeded (2 x 10^3^) into a 96-well plate. After overnight incubation, cells were transfected with either the ATF3 expressing or control vector using Xfect™ Transfection reagent (Clontech), following manufacturer’s instruction. After 48 h, media was refreshed, and 0.16, 0.31, 0.63 or 1.25 μM camptothecin was added where indicated. After 48 h of camptothecin treatment, cell viability was assessed using CellTiter-Blue® reagent (Promega) and the absorbance signal was measured by SpectraMax M2 plate reader (Molecular Devices, Sunnyvale, CA).

### Immunoblotting

Approximately 1x10^5^ SKOV-3 cells were seeded in a 6-well plate prior to transfection. Cells were harvested after 48 h priors to being lysed in radioimmunoprecipitation assay buffer. Proteins were separated by SDS-PAGE, transferred to polyvinylidene difluoride membrane and immunoblotted using a primary antibody against ATF3 (Santa Cruz Biotechnology, Inc., Dallas, TX), GAPDH or Actin (Cell Signaling Technology, Inc., Danvers, MA, USA), and an HRP-conjugated secondary antibody (GE Healthcare, Atlanta, GA). The protein bands were then visualized with SuperSignal™ West Dura Extended Duration Substrate (Thermo Fisher Scientific) using a Fluorchem HD2 chemiluminescent imaging system (Protein Simple, Santa Clara, CA). Protein bands were subsequently quantified using Image J (National Institutes of Health, Bethesda, MD) where indicated. Representative blots are shown and all experiments were repeated three times.

### Gene expression of patient specimens

No human subjects were directly involved in this research. The publicly available datasets, GSE9116 and datasets with expression of the NCI-60 cancer cell lines, were downloaded from the National Center for Biotechnology Information (NCBI) and mined for information relating to ATF3 or miR-30c-2-3p, respectively. Thus, all human identifiers are removed and the subjects are anonymous. Raw Affymetrix U133 Array data was plotted as a column scatter graph to visualize the distribution of the transcription factor data. Hierarchical clustering analysis and data visualization of miR-30c-2-3p was performed as previously described [11].

### Statistical analysis

GraphPad Prism was utilized to analyze statistical differences using an analysis of variance (ANOVA) test, followed by Bonferroni’s multiple comparison tests between groups. For comparisons of two groups, the Student’s t-test was used. Where it is shown in figures, *p < 0.05 **p < 0.01 and ***p < 0.001 indicate the levels of significance.

## Results

**Fig 1:**
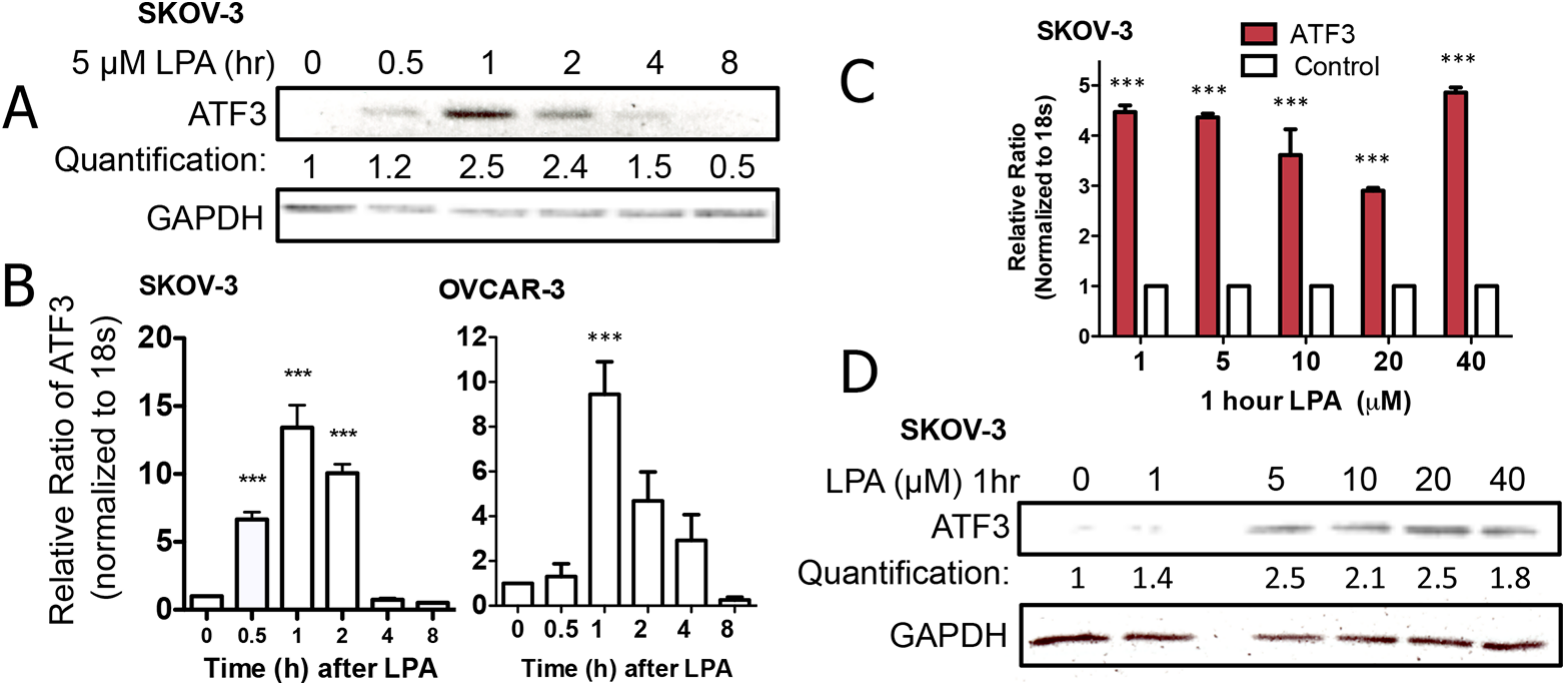
Lysophosphatidic acid induces the expression of ATF3. (A) SKOV-3 cells were treated with 5 μM lysophosphatidic acid (LPA) for the times indicated prior to assessment of ATF3 protein. (B) SKOV-3 and OVCAR-3 cells were treated with 5 μM LPA for the times indicated prior to qRT-PCR measurement and normalization of ATF3 transcripts. SKOV-3 cells were treated with increasing concentrations of LPA for 1 h before measuring ATF3 mRNA (C) or protein (D). ***p<0.001, comparing the indicated condition to time 0 or untreated control.

To commence our investigation, we added lysophosphatidic acid (5 μM) to SKOV-3 ovarian cancer cells and measured an increase in ATF3 protein levels (**Fig 1A**) and ATF3 mRNA transcripts (**Fig 1B**), over 8 h. Since ATF3 expression peaked 1 h after lysophosphatidic acid treatment, we also assessed a range of concentrations (1 to 40 μM) at the 1 h time point and found 5–20 μM to be effective in stimulating ATF3 mRNA transcription (**Fig 1C**) and ATF3 protein expression (**Fig 1D**) in SKOV-3 cells. Thus, we selected 5 μM for the remainder of the study because it is the lowest concentration that produces the response. Taken together, this data suggests that in ovarian cancer cells, lysophosphatidic acid stimulation induces the expression of ATF3.

**Fig 2:**
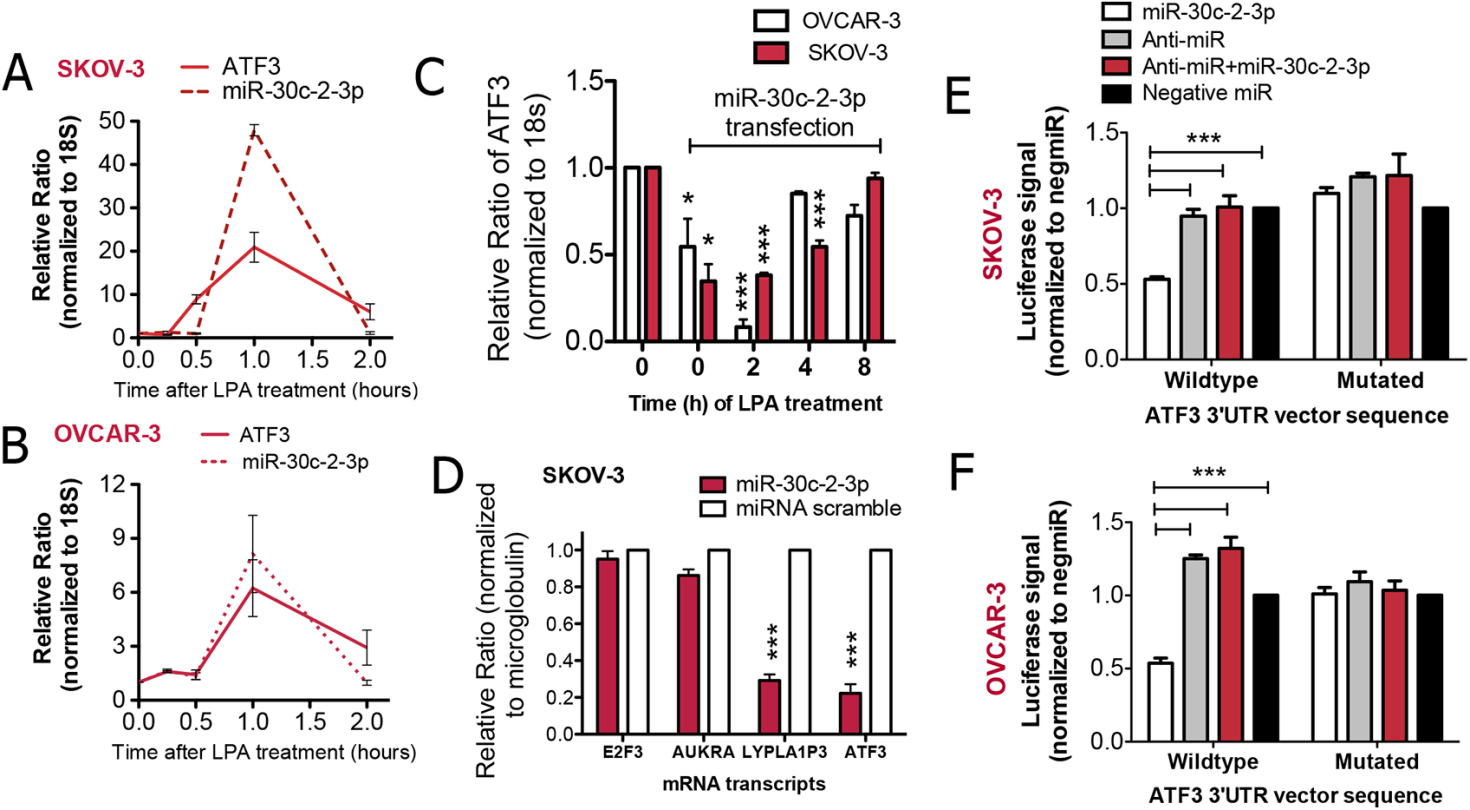
MiR-30c-2-3p inhibits ATF3 expression. (A) SKOV-3 or (B) OVCAR-3 cells were treated with lysophosphatidic acid (LPA, 5 μM) for 0.5, 1 or 2 h prior to the assessment of ATF3 and miR-30c-2-3p expression. The data is presented as a line graph of the relative ratio. (C) Cells were transfected with miRNA scramble or miR-30c-2-3p for 24 h prior to treatment with lysophosphatidic acid (LPA, 5 μM) for the times indicated and assessment of ATF3 expression. The bar graph shows the average relative ratio normalized to a housekeeping control. ***p<0.001, *p<0.05 (D) SKOV-3 cells were transfected with miR-30c-2-3p or a scramble miRNA control for 48 h prior to the quantification of mRNA transcripts. (E) SKOV-3 or (F) OVCAR-3 cells stably expressing an ATF3 3´UTR encoded luciferase vector (wild type) or a vector with mutations in the 3´UTR sequence (mutated) were transfected with either miR-30c-2-3p, anti-miR-30c-2-3p (anti-miR), miR-30c-2-3p + anti-miR-30c-2-3p or a control (negative-target miRNA) for 48 h prior to luciferase signal intensity measurement. ***p<0.001.

Intriguingly, a miR-30c-2-3p binding site was predicted for ATF3 using miRWalk 2.0 (**S2 Fig**). Indeed, lysophosphatidic acid elicits the expression of miR-30c-2-3p and ATF3, which both peak at 1 h (**Fig 2A and 2B**). When we introduced miR-30c-2-3p into cells and after 48 h, stimulated with lysophosphatidic acid, there was a significant reduction in ATF3 transcripts at 0, 2 and 4 h, which was absent by 8 h (**Fig 2C**). To confirm, we examined miR-30c-2-3p’s ability to suppress various mRNA expression after 48 h of introduction into cells. We observed no change among unrelated *E2F3* or *AUKRA* transcripts, but did observe a decrease in the expression of the specific targets, *ATF3* and *LYPLA1P3*, the latter which is lysophospholipase I pseudogene 3, a pseudogene located downstream of the miR-30c-2-3p gene on chromosome 6q13 (**Fig 2D**).

We therefore wanted to confirm that miR-30c-2-3p could bind to the 3´-untranslated region of ATF3. After stably expressing a luciferase vector in ovarian cancer cells that encoded the ATF3 3´-untranslated region sequence, we observed a significant reduction in luciferase among SKOV-3 cells transfected with miR-30c-2-3p, but not those also transfected with the neutralizing anti-miR-30c-2-3p (**Fig 2E**). However, a vector with specific mutations in the sequence did not reduce luciferase. The data was also repeated in experiments with OVCAR-3 cells (**Fig 2F**). This suggests that miR-30c-2-3p is binding to the 3´-untranslated region of ATF3.

**Fig 3:**
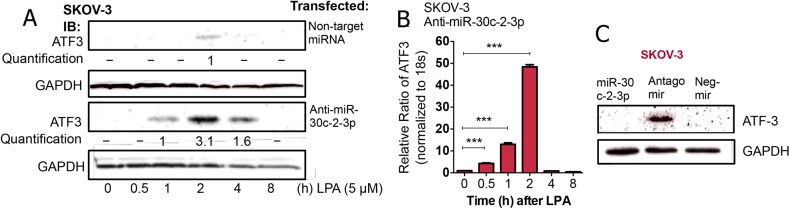
Anti-miR-30c-2-3p augments ATF3 expression. (A) SKOV-3 cells were transfected with either non-targeting-miRNA control or anti-miR-30c-2-3p, prior to treatment with LPA (5 μM) for the times indicated and visualization of ATF3 protein bands. (B) SKOV-3 cells were transfected with anti-miR-30c-2-3p for 48 h and then treated with lysophosphatidic acid for the times indicated prior to measuring ATF3 mRNA expression using qRT-PCR, in comparison to non-targeting-miRNA control. ***p<0.001 (C) ATF3 protein was measured among unstimulated SKOV-3 cells transfected for 48 h with either miR-30c-2-3p, anti-miR-30c-2-3p or a negative, non-targeting-miRNA control.

As further confirmation that miR-30c-2-3p targets the mRNA ATF3 transcript, we exogenously introduced anti-miR-30c-2-3p into SKOV-3 cells and then measured ATF3 expression after treatment with lysophosphatidic acid. Compared to transfection with a non-targeting miRNA, anti-miR-30c-2-3p enhanced ATF3 protein levels of at 1, 2 and 4 h after stimulation with lysophosphatidic acid (**Fig 3A**). Even the magnitude of ATF3 mRNA expression with anti-miR-30c-2-3p transfection was altered nearly 5-fold (**Fig 3B**), in comparison with our previous results (**Fig 1B**) and the duration shifted as well, so that the expression peaked at 2 h. Similarly, ATF3 protein was increased after anti-miR-30c-2-3p expression in untreated cells, compared with miR-30c-2-3p expression and a non-targeting control (**Fig 3C**). Taken together, this supports our hypothesis, which suggests that miR-30c-2-3p directly silences ATF3 expression.

**Fig 4:**
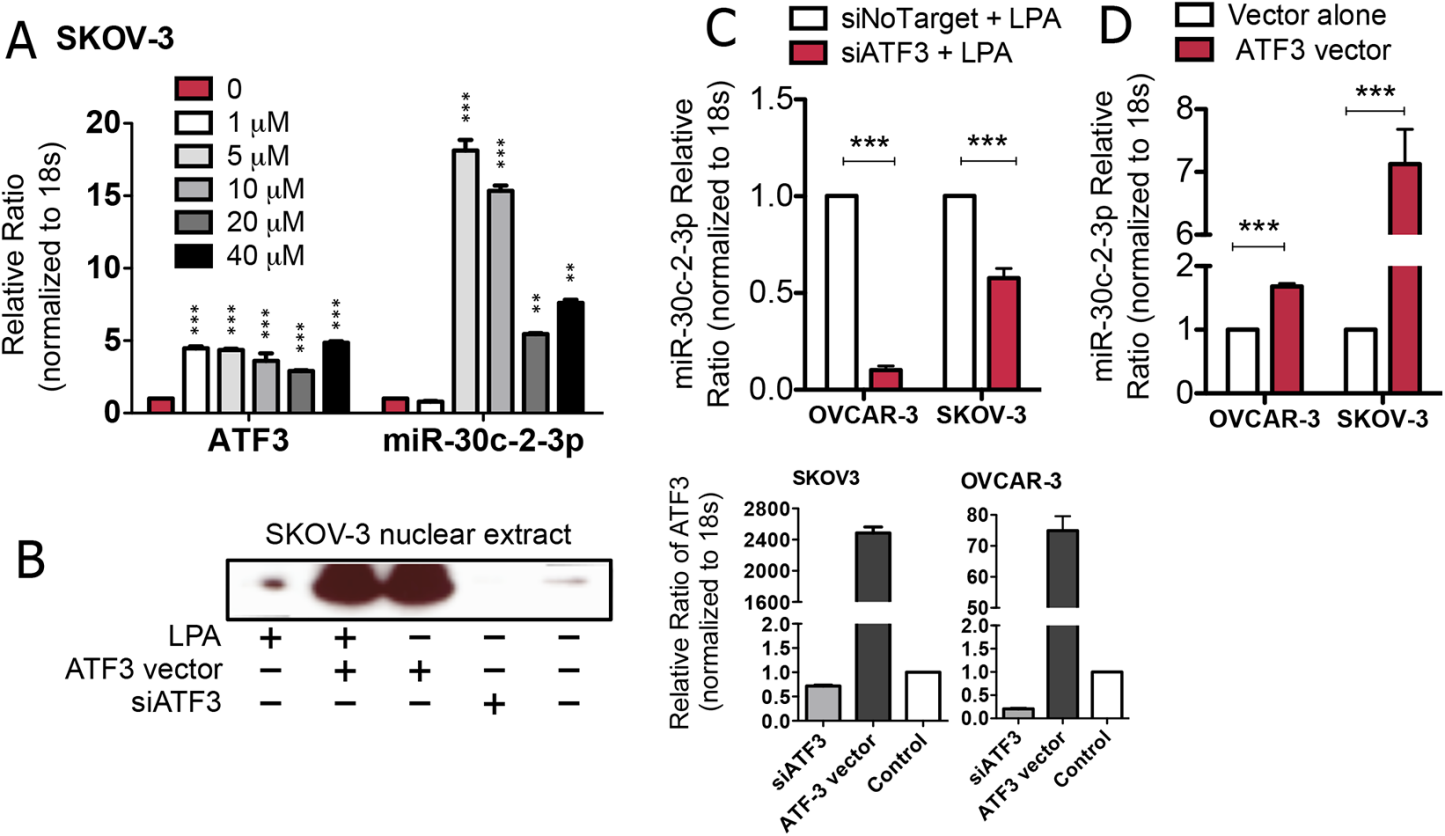
ATF3 modulates miR-30c-2-3p expression. (A) SKOV-3 cells were treated with increasing concentrations of lysophosphatidic acid (LPA) as indicated in μM for 1 h before measuring ATF3 mRNA and miR-30c-2-3p. (B) SKOV-3 cells were processed for immunoblotting to demonstrate the ability of the ATF3 expression vector and siATF3 to modulate ATF3 protein. Similarly, siATF3 enforced expression demonstrates the ability of the siRNA to suppress ATF3 mRNA transcription in SKOV-3 and OVCAR-3 cells using qRT-PCR normalized to microglobulin. (C) OVCAR-3 and SKOV-3 cells were transfected with siATF3 or an siNon-targeting control for 48 h prior to stimulation with LPA (5 μM, 1 h), followed by quantification of miR-30c-2-3p. (D) OVCAR-3 and SKOV-3 cells were transfected with an ATF3 expression vector or a control vector for 48 h prior to quantification of miR-30c-2-3p. ***p<0.001.

Treating cells with lysophosphatidic acid shows that the expression of both ATF3 and miR-30c-2-3p is increased (**Fig 4A**). However, we wanted to examine the inverse relationship and thereby determine whether the presence of ATF3 affects the expression of miR-30c-2-3p. Therefore, we suppressed ATF3, using siATF3, or increased ATF3 with an expression vector (**Fig 4B**), and stimulated SKOV-3 and OVCAR-3 cells with lysophosphatidic acid (5 μM for 1 h) for comparison of miR-30c-2-3p levels to cells without ATF3 suppression. Indeed, when ATF3 is reduced, even cells stimulated with lysophosphatidic acid display significant inhibition of miR-30c-2-3p transcript expression (**Fig 4C**). In contrast, when cells were transfected with the ATF3 vector to increase the expression/function of the transcription factor, both cell lines increased the expression of miR-30c-2-3p (**Fig 4D**), especially SKOV-3, without requiring lysophosphatidic acid treatment. Taken together, these data suggest that ATF3 affects the expression of the miR-30c-2-3p transcript.

**Fig 5:**
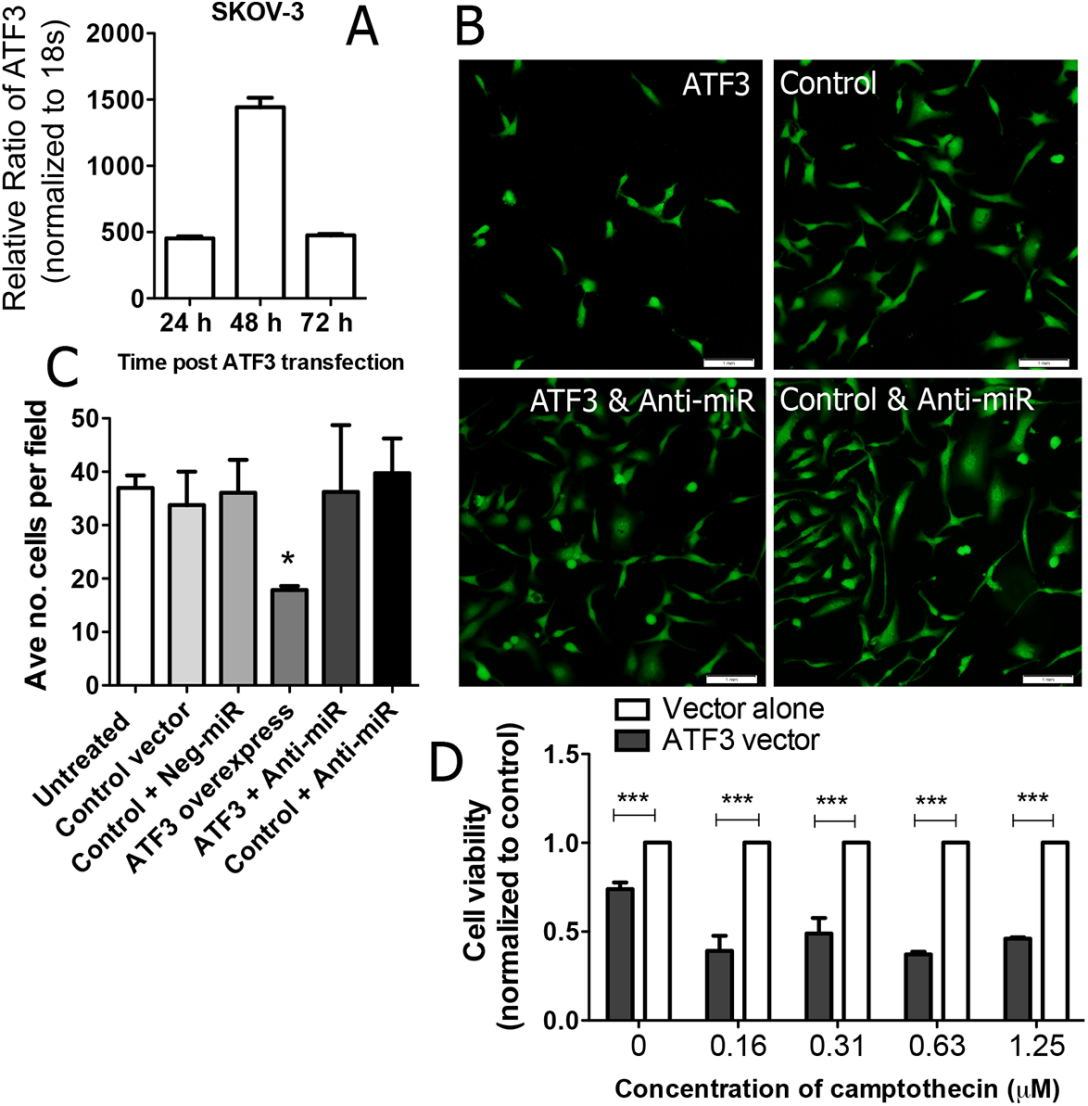
ATF3 expression induces cellular stress on ovarian cancer cells. (A) ATF3 mRNA expression was measured in SKOV-3 cells after 24, 48 and 72 h of transfection with an ATF3 expression vector. (B) Untreated SKOV-3 cells were transfected as indicated with a combination of either ATF3 expression vector, control vector or anti-miR-30c-2-3p, and then stained for fluorescence imaging using a whole cell marker. (C) High-throughput, automatic quantification software counted ~10 fields of SKOV-3 cells (equivalent to 150–750 cells in total) from the previous experiment. *p<0.05, comparing ATF3 expression vs. control, untreated or control + negative-miR conditions. (D) Cell viability was measured in SKOV-3 cells after ATF3 over-expression compared with control vector after treatment with the indicated concentrations of camptothecin. ***p<0.001.

In previous work, we established that miR-30c-2-3p inhibits cell proliferation [6]. Therefore, the expression of ATF3 should have the same effect, if it induces miR-30c-2-3p expression. To test this prediction, we transfected ATF3 into SKOV-3 cells (**Fig 5A**) and observed a striking reduction in the number of cells under fluorescence microscopy using whole cell staining (**Fig 5B**). We quantified this observation using automated, high-throughput ArrayScan imaging to assess the number of cells among >10 fields (approximately 150–750 cells total) and detected a significant reduction upon over-expression of ATF3 (**Fig 5C**, *p<0.05). Correspondingly, the effect was blocked by anti-30c-2-3p. This data suggests a similar functional outcome on proliferation between miR-30c-2-3p and ATF3. Lastly, since ATF3 can enhance cellular stress, ATF3 was transfected into SKOV-3 cells before treatment with campothecin, wherein we observed a significant reduction in the cell viability (**Fig 5D**). Although this may not be the only mechanism responsible, it does suggest that the ability of ATF3 to negatively affect cell proliferation can occur via miR-30c-2-3p transcription.

## Discussion

**Fig 6:**
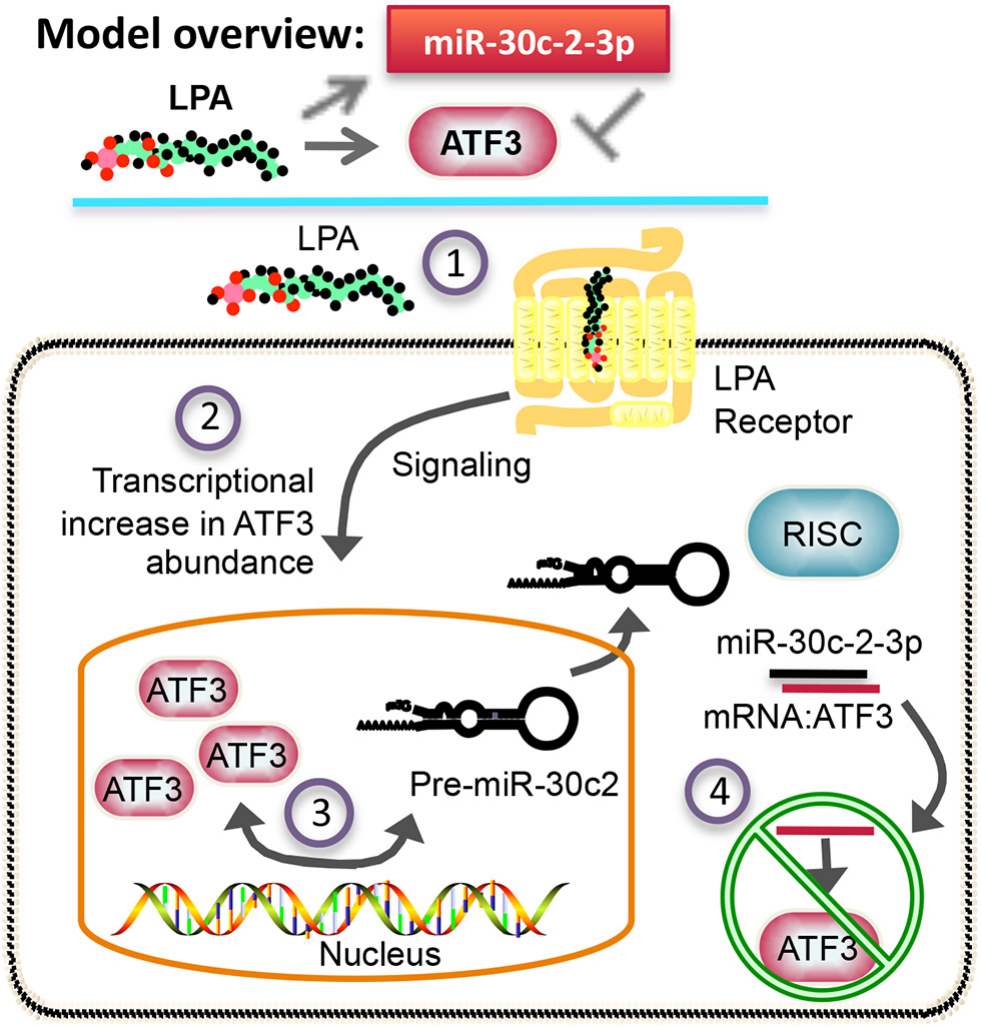
Model of auto-regulatory feedback loop between ATF3 and miR-30c-2-3p. (1) Receptor signaling mediated by lysophosphatidic acid (LPA) (2) induces ATF3 expression. (3) The expression of pre-miR-30c-2 also increases, along with ATF3. (4) Processing leads to miR-30c-2-3p transcripts and subsequent binding to ATF3, causing silencing of further ATF3 expression. (Note: model does not accurately depict proper localization of molecules.)

Based on our experimental data, we propose that in ovarian cancer cells, lysophosphatidic acid mediates the expression of both the transcription factor ATF3 and miR-30c-2-3p, which is a miRNA primarily expressed in ovarian and renal cancers (**S3 Fig**) that we have previously shown is also induced by lysophosphatidic acid [6]. As a result, miR-30c-2-3p binds to the 3´-untranslated region of ATF3 and reduces mRNA and protein expression. Taken together, the data suggests the current working molecular model for this system (**Fig 6**). This pathway ultimately leads to the decline of both molecules, the transcription factor and miRNA, which were initially expressed as a result of lysophosphatidic acid signaling.

While the tumorigenic effects of lysophosphatidic acid are well known [10, 11, 25–28], which also involve the highly-researched aspects of G protein-coupled receptor signaling, autotaxin and phosphatase enzymes, the molecular mechanisms beyond the cell surface are less appreciated. To date, there are only a handful of papers examining miRNA mechanisms associated with lysophosphatidic acid; correspondingly, our previous report on miR-30c-2-3p was the first in the literature in this area [6]. Thus, our study herein fills a major gap in the molecular understanding of signaling circuits initiated by lysophosphatidic acid, especially at the level of post-transcriptional silencing regulated by miRNA. After characterizing the miRNA response to lysophosphatidic acid-mediated signaling [6], we now further dissect the molecular mechanism and outcomes through the inhibition of the transcription factor, ATF3.

Although this is a novel mechanism demonstrating that miR-30c-2-3p inhibits ATF3, the literature reports the existence of negative feedback loops between miRNA and their targets. For example, miR-200a, miR-200b and miR-429 comprise a double-negative feedback loop with the transcription repressors ZEB1 and SIP1 in breast cancer cells [29]. Another example of a double-negative feedback loop system occurs with miR-422a and three of its targets, the forkhead box genes, which regulate the development of hepatocellular carcinoma [30]. Positive, auto-regulatory feedback loops also exist, for example, between miR-448 and NF-κB in breast cancer cells [31]. In addition, another study uncovered a regulatory axis in a tightly linked transcriptional system between p55PIIK, p53 and miR-148b in colorectal cancer cells [30]. Thus, our data parallels these studies in that regulatory mechanisms exist between transcription factors and miRNA expression.

Furthermore, the study herein provides experimental data to support the regulation of another gene transcript targeted via miR-30c-2-3p. To date, this list now includes ATF3, BCL9, HIF2A, X-box binding protein 1, Cyclin E1 and an adaptor protein of the NF-κB signaling pathway [6, 20, 32, 33]. The fact that many of the aforementioned are known oncoproteins and/or involved in cell proliferation is consistent with our data. We show that the overexpression of ATF3 significantly reduces the number of ovarian cancer cells, which suggests an influence on proliferation–a hallmark of cancer [34].

Since epigenetics are involved in the development and progression of ovarian cancer [35, 36], we initially hypothesized that miR-30c-2-3p could be induced by alterations in DNA methylation or histone acetylation. However, we did not observe consistent increases in miR-30c-2-3p expression after treating cells with epigenetic modifiers, which would have indicated a reactivation of silenced genes. For example, treatment with 5-azacytidine and/or 3-Deazaneplanocin A over a period of 1–11 days did not exhibit a similar and/or substantial increase in miR-30c-2-3p that is observed upon stimulation with 5 μM lysophosphatidic acid after 1–2 h (data not shown), which would have emulated our previous results (**Fig 1A and 1B**). Thus, epigenetic mechanisms could not explain the considerable activation of miR-30c-2-3p that would also account for the transient nature observed here.

Both ATF3 and miR-30c-2-3p are detected at negligible levels among quiescent cells and are dramatically increased after 1 h of lysophosphatidic acid stimulation. Furthermore, if miRNAs were globally reduced among cancer cells [2, 4], then this would theoretically indicate the possibility of enhanced activity by ATF3 in such a system, which could exacerbate disproportionate protein expression. However, the fate of miR-30c-2-3p under such a scenario is yet to be determined experimentally. Future studies will focus on this area.

## Supporting Information

S1 Fig ATF3 gene transcription is increased among primary ovarian cancer tumors among patients with a high level of depression. The GEO Dataset, GSE9116 [14, 15], was downloaded from the NCBI and mined for the expression of transcription factors that have a relationship with lysophosphatidic acid signaling in ovarian cancer [11]. Although EPAS1, ETV5 and SKIL were not significant, the difference in ATF3 raw gene expression between patients with high and low depression was significant. **p<0.01
(TIF)

S2 Fig Schematics of the interaction. The sequence of miR-30c-2-3p is shown as well as a schematic representation of the 3´-untranslated region of ATF3 with the predicted target site for miR-30c-2-3p highlighted in yellow. For the luciferase experiments using the mutated vector, the sequence is also presented.
(TIF)

S3 Fig MiR-30c-2-3p is predominantly expressed in ovarian and renal cancer cell lines. Gene expression data from the NCI-60 set of cell lines was downloaded and mined for the expression of miR-30c-2-3p. The highest expression was detected mainly among two cell types: ovarian (OV) and renal (RE). Other abbreviations among the cell lines in the NCI-60 include breast (BR), central nervous system (CNS), colon (CO), leukemia (LE), lung (LC), melanoma (ME) and prostate (PR). The range of logarithmic expression is from 2.685 (red) to -1.345 (green).
(TIF)
