# Supplementary figures and images for: Lysophosphatidic Acid Mediates Activating Transcription Factor 3 Expression Which Is a Target for Post-Transcriptional Silencing by miR-30c-2-3p
Source: PLoS One. 2015 Sep 29;10(9):e0139489. doi: 10.1371/journal.pone.0139489 (PMC4587950; doi:10.1371/journal.pone.0139489)

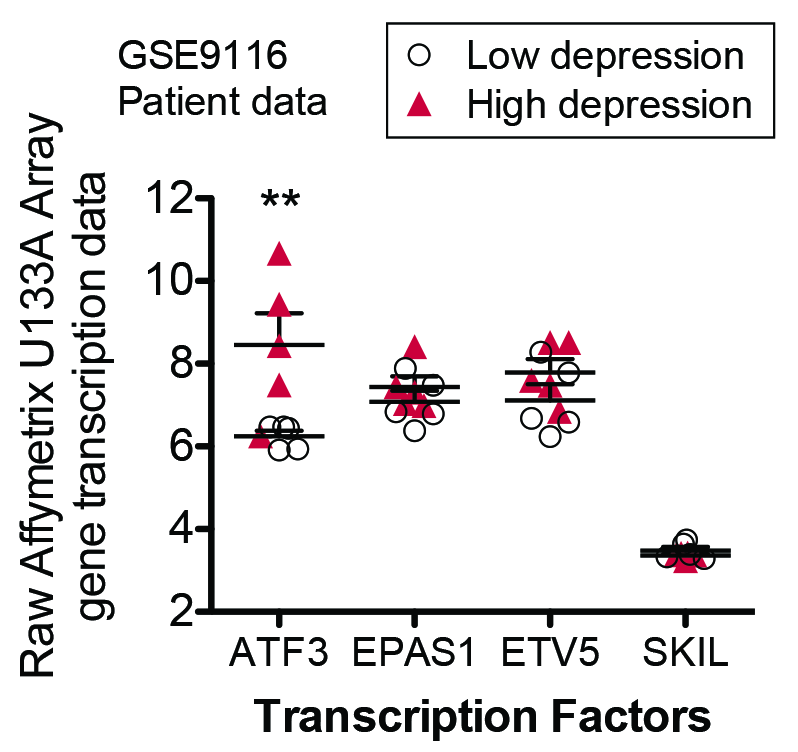

Supplement: S1 Fig — The GEO Dataset, GSE9116 [14, 15], was downloaded from the NCBI and mined for the expression of transcription factors that have a relationship with lysophosphatidic acid signaling in ovarian cancer [11]. Although EPAS1, ETV5 and SKIL were not significant, the difference in ATF3 raw gene expression between patients with high and low depression was significant. **p<0.01 (TIF) [file pone.0139489.s001.tif]

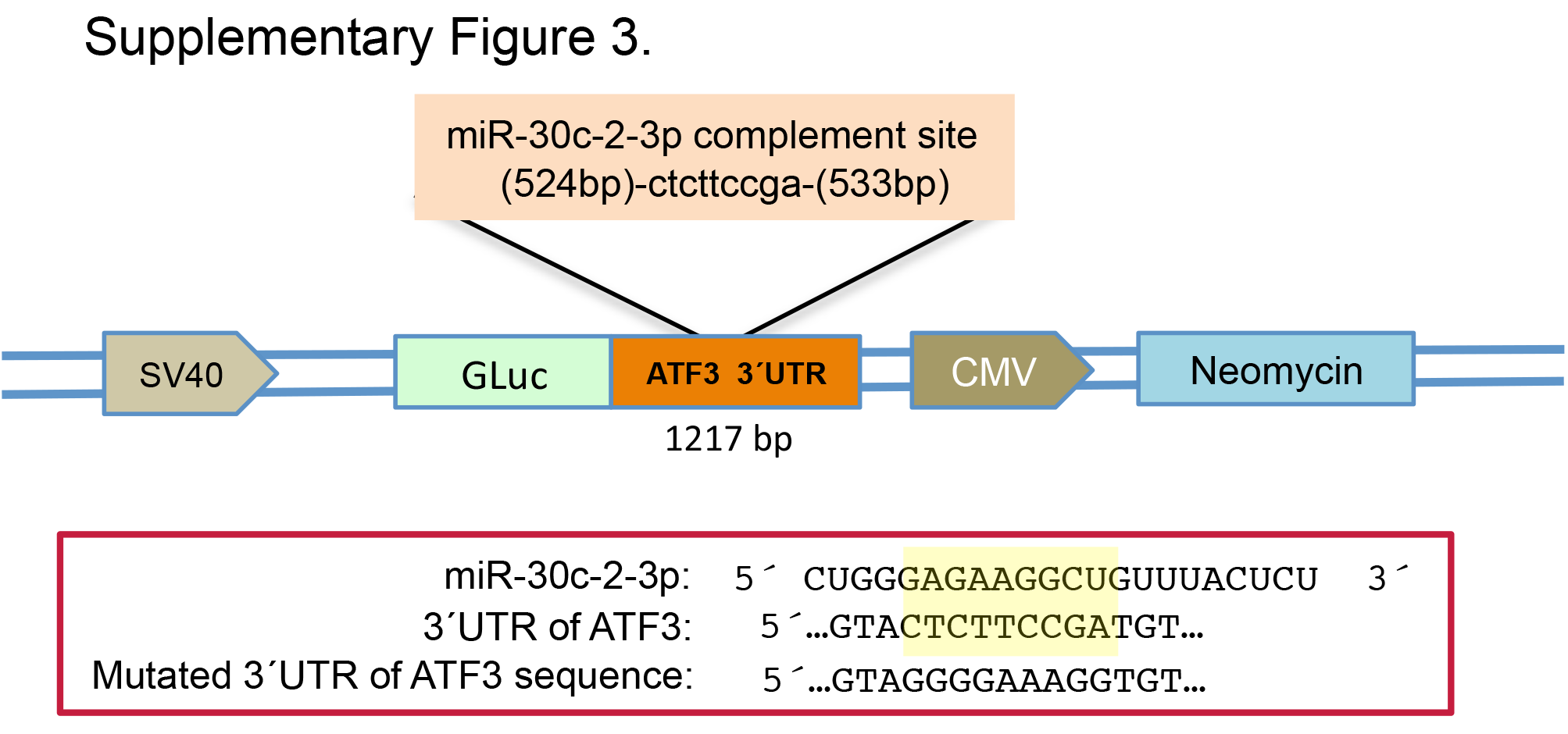

Supplement: S2 Fig — The sequence of miR-30c-2-3p is shown as well as a schematic representation of the 3´-untranslated region of ATF3 with the predicted target site for miR-30c-2-3p highlighted in yellow. For the luciferase experiments using the mutated vector, the sequence is also presented. (TIF) [file pone.0139489.s002.tif]

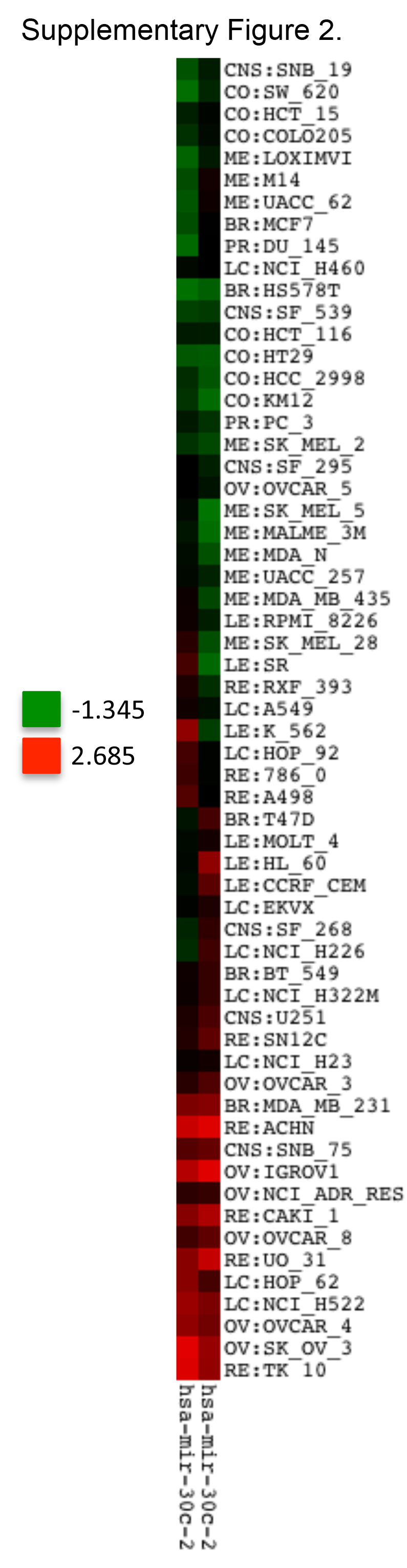

Supplement: S3 Fig — Gene expression data from the NCI-60 set of cell lines was downloaded and mined for the expression of miR-30c-2-3p. The highest expression was detected mainly among two cell types: ovarian (OV) and renal (RE). Other abbreviations among the cell lines in the NCI-60 include breast (BR), central nervous system (CNS), colon (CO), leukemia (LE), lung (LC), melanoma (ME) and prostate (PR). The range of logarithmic expression is from 2.685 (red) to -1.345 (green). (TIF) [file pone.0139489.s003.tif]
